# Supplementary material for: Microbial sensor variation across biogeochemical conditions in the terrestrial deep subsurface
Source: mSystems. 2023 Dec 7;9(1):e00966-23. doi: 10.1128/msystems.00966-23 (PMC10805038; doi:10.1128/msystems.00966-23)
Supplement: Supporting figures and tables — Figures S1-S8; Tables S1-S3. [file msystems.00966-23-s0004.docx]

**SUPPORTING INFORMATION**

**Microbial sensor variation across biogeochemical conditions in the terrestrial deep subsurface**

Annelise L. Goldman^a,†,*^, Emily M. Fulk^b,†,‡^, Lily Momper^c,¶^, Clinton Heider^d^, John Mulligan^d^, Magdalena Osburn^c^, Caroline A. Masiello^a,e,f^, and Jonathan J. Silberg^a,g,h^#

Author affiliations:

^a^Department of Biosciences, Rice University, Houston, TX, USA

^b^Systems, Synthetic, and Physical Biology Graduate Program, Rice University, Houston, TX, USA

^c^Department of Earth and Planetary Sciences, Northwestern University, Evanston, IL, USA

^d^Center for Research Computing, Rice University, Houston, TX, USA

^e^Department of Earth, Environmental and Planetary Sciences, Rice University, Houston, TX, USA

^f^Department of Chemistry, Rice University, Houston, TX, USA

^g^Department of Bioengineering, Rice University, Houston, TX, USA

^h^Department of Chemical and Biomolecular Engineering, Rice University, Houston, TX, USA

^†^these authors contributed equally to this manuscript (order determined via discussion)

# Address Correspondence to Jonathan J. Silberg, Department of Biosciences, Rice University, 6100 Main Street, MS-140, Houston, TX, 77005; Tel: 713-348-3849; Email: [joff@rice.edu](mailto:joff@rice.edu)

^*^Present address: Department of Plant and Microbial Biology, University of California Berkeley, CA, USA.

^‡^Present address: National Renewable Energy Laboratory, Golden, CO, USA.

^¶^Present address: Exponent, Pasadena, CA, USA.

**Running title:** Microbial sensor variation within the deep subsurface.

**KEYWORDS**: dissolved organic carbon, geochemistry, histidine kinase, microbe, mine, response regulator, subsurface, sensor, two-component systems


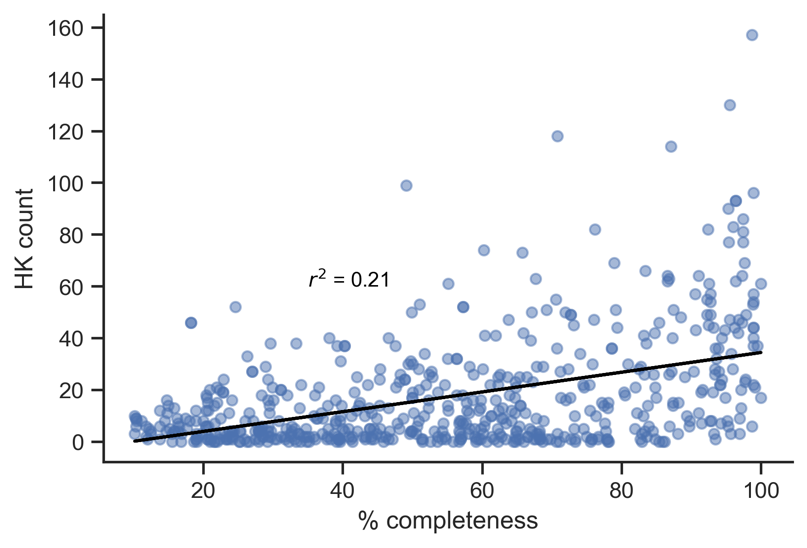


**Figure S1. Relationship between HK abundances in each MAG and the percentage MAG completeness.** For all MAGs in the dataset, the absolute number of HKs is plotted versus the % completeness previously reported (24). A linear fit yields a weak correlation (r^2^ = 0.21), suggesting that the variation in HK abundances across the different MAGs is not determined by MAG completeness.


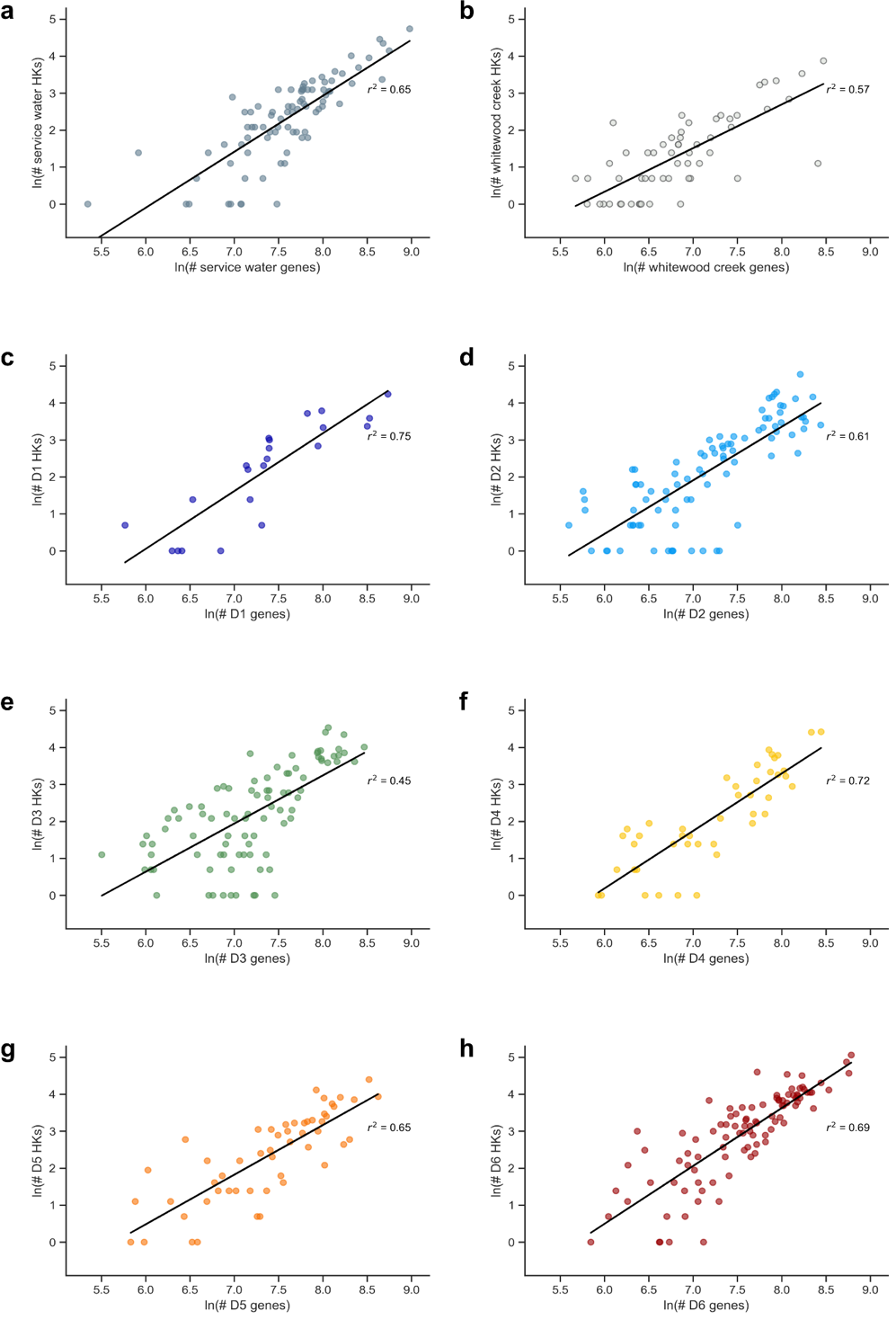


**Figure S2. HK abundance correlates with genome size at each sampling site.** The number of total genes and genes encoding HKs is shown for every MAG at: (**a**) the service water, (**b**) Whitewood Creek, (**c**) D1, (**d**) D2, (**e**) D3, (**f**) D4, (**g**) D5, and (**h**) D6 sites. The r^2^ values from linear regression are shown in each plot, indicating the extent to which each log-log plot follows a linear trend. MAGs lacking HKs were removed from the dataset to facilitate plotting. The x-axis data were derived by taking the natural log of the number of predicted proteins. The data shows that HK abundance presents a power law relationship with genome size.

**Figure S3. Comparison of the minimum biodiversity values and HK frequencies.** At each sampling site, the biodiversity was quantified at different time points using rRNA sequencing data acquired over fourteen sample collections between 2015 and 2019. For each sampling site, the minimum values obtained for each diversity metric from all of these sample collections is plotted versus the HK frequencies observed from a single sample collection in 2018. Diversity metrics included the minimum values for: (**a**) the Chao1 index, (**b**) the number of OTUs, (**c**) the phylogenetic distance, (**d**) the Shannon Index, (**e**) the Simpson Index, and (**f**) the Simpson Evenness.

**Figure S4. Comparison of the maximum biodiversity values with HK frequencies.** At each sampling site, the biodiversity was quantified at different time points using rRNA sequencing data acquired over fourteen sample collections between 2015 and 2019. For each sampling site, the maximum values obtained for each diversity metric from all of these sample collections is plotted versus the HK frequencies observed from a single sample collection in 2018. Diversity metrics included the maximum values for: (**a**) the Chao1 index, (**b**) the number of OTUs, (**c**) the phylogenetic distance, (**d**) the Shannon Index, (**e**) the Simpson Index, and (**f**) the Simpson Evenness.

**Figure S5. Comparison of the mean biodiversity values with HK frequencies.** At each sampling site, the biodiversity was quantified at different time points using rRNA sequencing data acquired over fourteen sample collections between 2015 and 2019. For each sampling site, the mean values obtained for each diversity metric from all of these sample collections is plotted versus the HK frequencies observed from a single sample collection in 2018. Diversity metrics included the mean values for: (**a**) the Chao1 index, (**b**) the number of OTUs, (**c**) the phylogenetic distance, (**d**) the Shannon Index, (**e**) the Simpson Index, and (**f**) the Simpson Evenness.


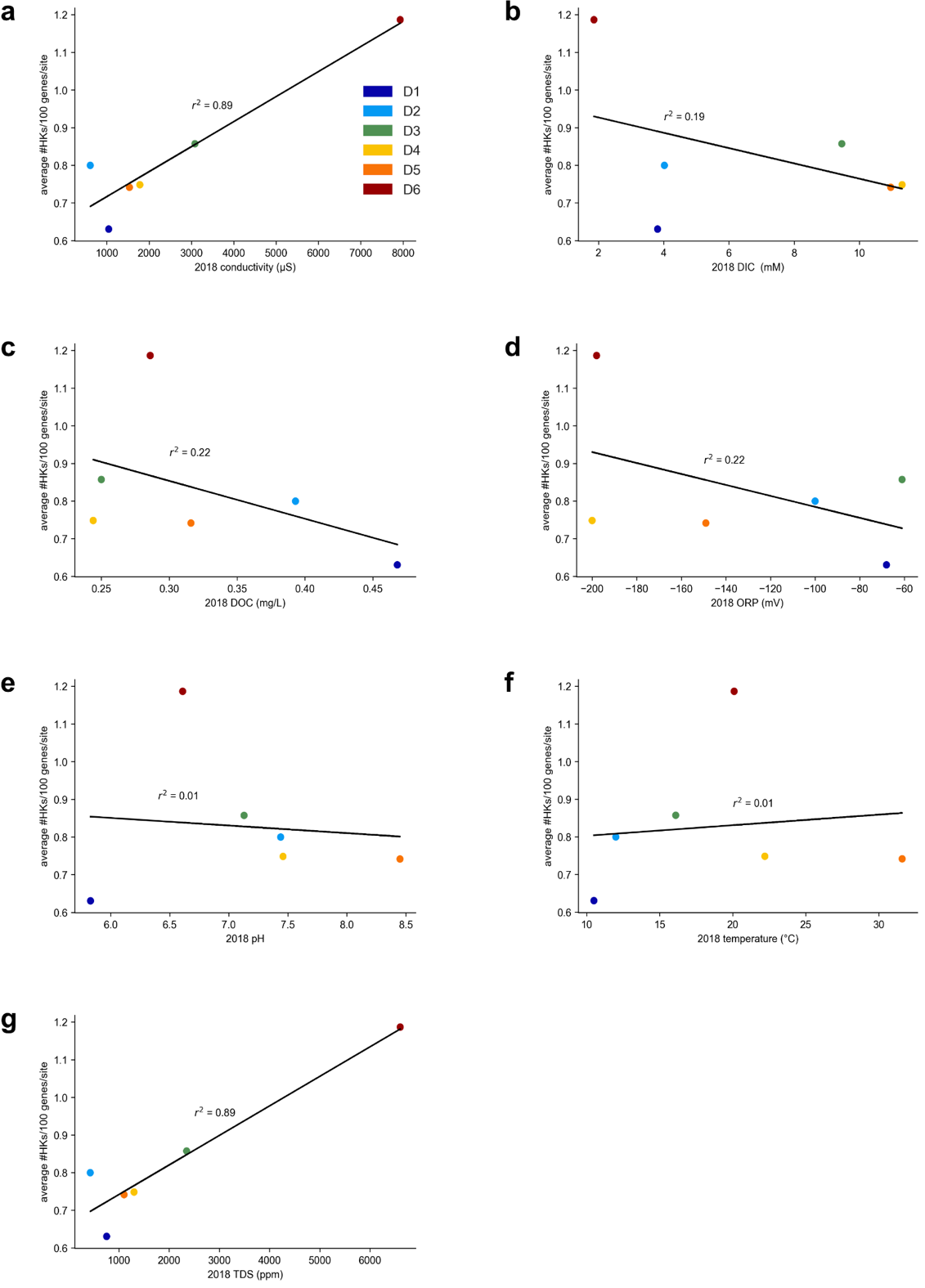


**Figure S6. Relationships between complex geochemical properties and HK frequencies at a single time point.** At each sampling site, geochemical parameters were measured at the same time as the MAGs were collected in 2018. For each site, the measured parameter is shown versus the calculated HK frequency. The properties compared include: (**a**) conductivity (µS), (**b**) dissolved inorganic carbon (DIC, mM), (**c**) dissolved organic carbon (DOC, mM), (**d**) oxidation reduction potential (ORP, mV), (**e**) pH, (**f**) temperature (°C), and (**g**) total dissolved solids (TDS, ppm).

**
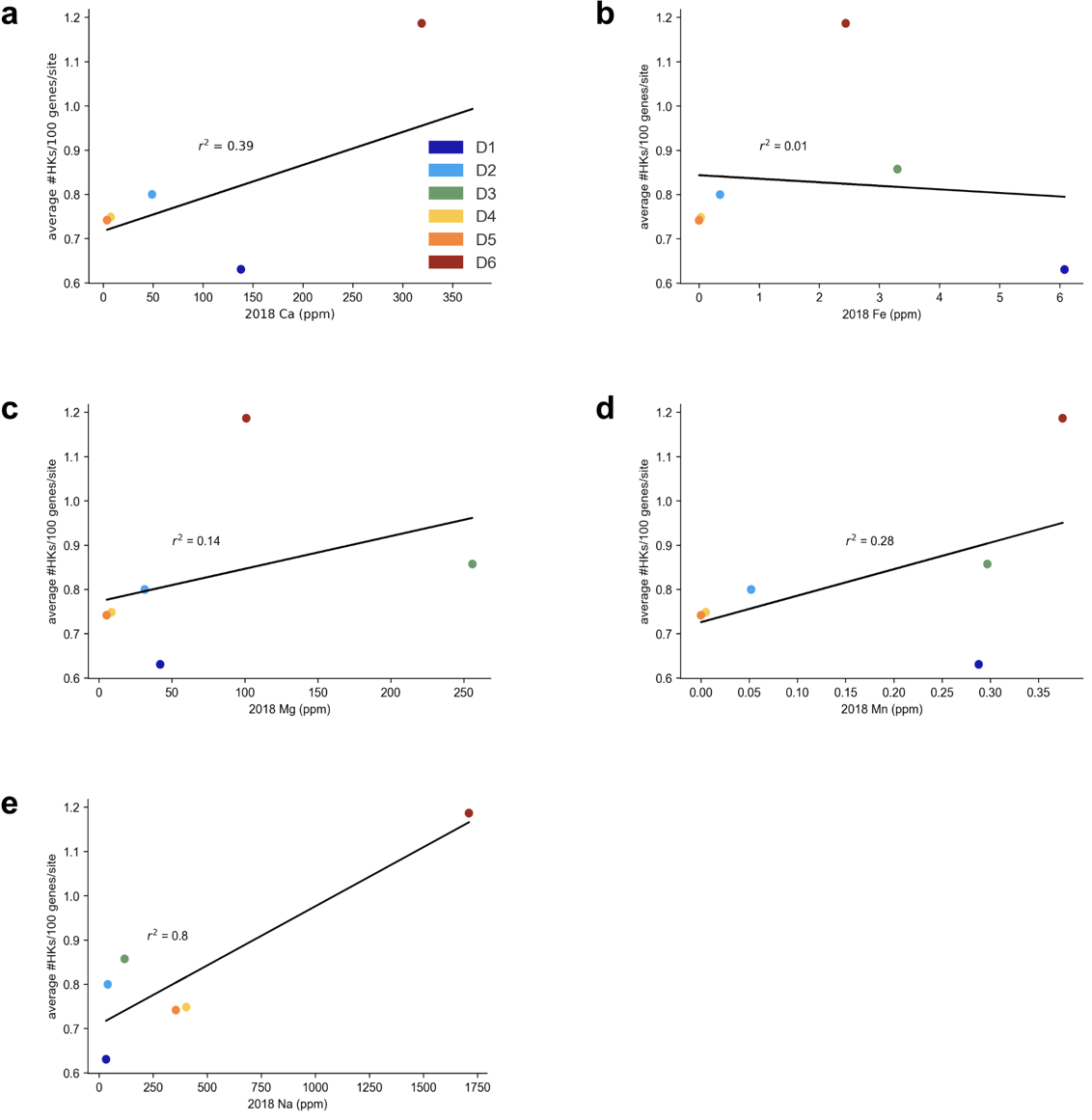
**

**Figure S7. Relationships between metal abundances and HK frequencies at a single time.** At each sampling site, (**a**) Ca (ppm), (**b**) Fe (including both ions, ppm), (**c**) Mg (ppm), (**d**) Mn (ppm), and (**e**) Na (ppm) concentrations were measured at the same time as MAGs were collected in 2018. The concentrations are shown here versus calculated HK frequency at each site.


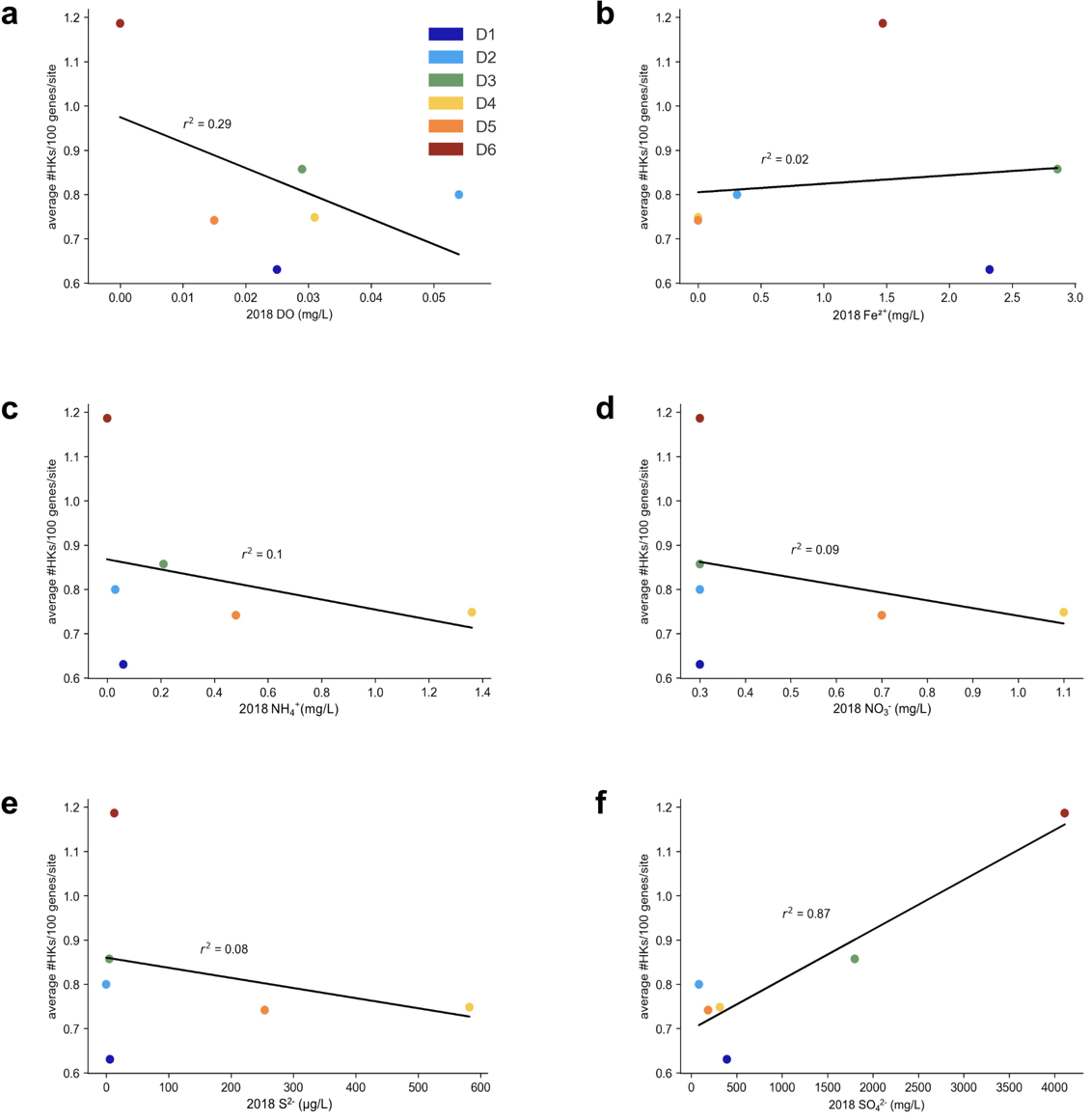


**Figure S8. Relationships between redox molecules and HK frequencies at a single time.** At each sampling site, (**a**) dissolved oxygen (DO, mg/L), (**b**) Fe²⁺ (mg/L), (**c**) NH₄⁺ (mg/L), (**d**) NO₃⁻ (mg/L), (**e**) S²⁻ (μg/L), and (**f**) SO₄²⁻ (mg/L) were measured at the same time as MAGs were collected in 2018. The concentrations of each is shown versus the calculated HK frequency at each site. While H₂ is shown as a redox molecule in Figure 6, it was not measured at the same time as MAGs were collected, and thus is not shown.

**Table S1. Tukey-Kramer analysis of average HK frequency between phyla.** p values calculated using a post-hoc Tukey-Kramer test of average HK frequencies between phyla. Only phyla with n $\geq$ 6 are included. p<0.05 are highlighted in yellow.

|  | Actinobacteriota | Bacteroidota | Chloroflexota | Cyanobacteriota | Dependentiae | Desulfobacterota | Elusimicrobiota | Firmicutes_B | Firmicutes_E | Micrarchaeota | Nanoarchaeota | Nitrospirota | Omnitrophota | Patescibacteria | Planctomycetota | Proteobacteria | Thermoplasmatota | Verrucomicrobiota | Zixibacteria |
| --- | --- | --- | --- | --- | --- | --- | --- | --- | --- | --- | --- | --- | --- | --- | --- | --- | --- | --- | --- |
| Actinobacteriota | 1.000 | 0.471 | 0.302 | 0.900 | 0.012 | 0.001 | 0.900 | 0.900 | 0.870 | 0.004 | 0.010 | 0.014 | 0.900 | 0.009 | 0.900 | 0.900 | 0.900 | 0.782 | 0.713 |
| Bacteroidota | 0.471 | 1.000 | 0.900 | 0.900 | 0.001 | 0.001 | 0.900 | 0.900 | 0.001 | 0.001 | 0.001 | 0.900 | 0.001 | 0.001 | 0.087 | 0.001 | 0.025 | 0.001 | 0.900 |
| Chloroflexota | 0.302 | 0.900 | 1.000 | 0.900 | 0.001 | 0.001 | 0.900 | 0.893 | 0.001 | 0.001 | 0.001 | 0.900 | 0.001 | 0.001 | 0.044 | 0.001 | 0.014 | 0.001 | 0.900 |
| Cyanobacteriota | 0.900 | 0.900 | 0.900 | 1.000 | 0.001 | 0.042 | 0.900 | 0.900 | 0.137 | 0.001 | 0.001 | 0.900 | 0.170 | 0.001 | 0.900 | 0.894 | 0.432 | 0.087 | 0.900 |
| Dependentiae | 0.012 | 0.001 | 0.001 | 0.001 | 1.000 | 0.001 | 0.013 | 0.094 | 0.900 | 0.900 | 0.900 | 0.001 | 0.225 | 0.900 | 0.013 | 0.001 | 0.899 | 0.900 | 0.001 |
| Desulfobacterota | 0.001 | 0.001 | 0.001 | 0.042 | 0.001 | 1.000 | 0.052 | 0.001 | 0.001 | 0.001 | 0.001 | 0.561 | 0.001 | 0.001 | 0.001 | 0.001 | 0.001 | 0.001 | 0.248 |
| Elusimicrobiota | 0.900 | 0.900 | 0.900 | 0.900 | 0.013 | 0.052 | 1.000 | 0.900 | 0.622 | 0.009 | 0.014 | 0.900 | 0.810 | 0.044 | 0.900 | 0.900 | 0.890 | 0.563 | 0.900 |
| Firmicutes_B | 0.900 | 0.900 | 0.893 | 0.900 | 0.094 | 0.001 | 0.900 | 1.000 | 0.900 | 0.074 | 0.101 | 0.306 | 0.900 | 0.308 | 0.900 | 0.900 | 0.900 | 0.900 | 0.900 |
| Firmicutes_E | 0.870 | 0.001 | 0.001 | 0.137 | 0.900 | 0.001 | 0.622 | 0.900 | 1.000 | 0.900 | 0.900 | 0.001 | 0.900 | 0.900 | 0.900 | 0.684 | 0.900 | 0.900 | 0.014 |
| Micrarchaeota | 0.004 | 0.001 | 0.001 | 0.001 | 0.900 | 0.001 | 0.009 | 0.074 | 0.900 | 1.000 | 0.900 | 0.001 | 0.121 | 0.900 | 0.003 | 0.001 | 0.900 | 0.900 | 0.001 |
| Nanoarchaeota | 0.010 | 0.001 | 0.001 | 0.001 | 0.900 | 0.001 | 0.014 | 0.101 | 0.900 | 0.900 | 1.000 | 0.001 | 0.215 | 0.900 | 0.010 | 0.001 | 0.900 | 0.900 | 0.001 |
| Nitrospirota | 0.014 | 0.900 | 0.900 | 0.900 | 0.001 | 0.561 | 0.900 | 0.306 | 0.001 | 0.001 | 0.001 | 1.000 | 0.001 | 0.001 | 0.001 | 0.001 | 0.001 | 0.001 | 0.900 |
| Omnitrophota | 0.900 | 0.001 | 0.001 | 0.170 | 0.225 | 0.001 | 0.810 | 0.900 | 0.900 | 0.121 | 0.215 | 0.001 | 1.000 | 0.287 | 0.900 | 0.642 | 0.900 | 0.900 | 0.009 |
| Patescibacteria | 0.009 | 0.001 | 0.001 | 0.001 | 0.900 | 0.001 | 0.044 | 0.308 | 0.900 | 0.900 | 0.900 | 0.001 | 0.287 | 1.000 | 0.003 | 0.001 | 0.900 | 0.900 | 0.001 |
| Planctomycetota | 0.900 | 0.087 | 0.044 | 0.900 | 0.013 | 0.001 | 0.900 | 0.900 | 0.900 | 0.003 | 0.010 | 0.001 | 0.900 | 0.003 | 1.000 | 0.900 | 0.900 | 0.860 | 0.440 |
| Proteobacteria | 0.900 | 0.001 | 0.001 | 0.894 | 0.001 | 0.001 | 0.900 | 0.900 | 0.684 | 0.001 | 0.001 | 0.001 | 0.642 | 0.001 | 0.900 | 1.000 | 0.900 | 0.517 | 0.263 |
| Thermoplasmatota | 0.900 | 0.025 | 0.014 | 0.432 | 0.899 | 0.001 | 0.890 | 0.900 | 0.900 | 0.900 | 0.900 | 0.001 | 0.900 | 0.900 | 0.900 | 0.900 | 1.000 | 0.900 | 0.092 |
| Verrucomicrobiota | 0.782 | 0.001 | 0.001 | 0.087 | 0.900 | 0.001 | 0.563 | 0.900 | 0.900 | 0.900 | 0.900 | 0.001 | 0.900 | 0.900 | 0.860 | 0.517 | 0.900 | 1.000 | 0.006 |
| Zixibacteria | 0.713 | 0.900 | 0.900 | 0.900 | 0.001 | 0.248 | 0.900 | 0.900 | 0.014 | 0.001 | 0.001 | 0.900 | 0.009 | 0.001 | 0.440 | 0.263 | 0.092 | 0.006 | 1.000 |

**Table S2. Statistical analysis of HK correlations with biotic diversity metrics.** r^2^ and p values for the correlations calculated using least squares linear regression between mean alpha diversity metrics over time and HK frequency.

| **Alpha diversity metric** | **r^2^ value** | **p value** |
| --- | --- | --- |
| Chao1 | 0.55 | 0.056 |
| Number of OTUs | 0.59 | 0.044 |
| Phylogenetic distance | 0.58 | 0.047 |
| Shannon index | 0.81 | 0.006 |
| Simpson index | 0.74 | 0.014 |
| Simpson evenness | 0.03 | 0.711 |

**Table S3. Statistical analysis of HK correlations with abiotic properties.** r^2^ and p values for the correlations calculated using least squares linear regression between the standard deviation of geochemical parameters over time and HK frequency.

| **Geochemical parameter** | **r^2^ value** | **p value** |
| --- | --- | --- |
| DOC (mM) | 0.82 | 0.013 |
| Temperature (°C) | 0.57 | 0.082 |
| ORP (mV) | 0.42 | 0.162 |
| TDS (ppm) | 0.40 | 0.177 |
| pH | 0.36 | 0.209 |
| Conductivity (μS) | 0.28 | 0.284 |
| DIC (mM) | 0.02 | 0.797 |
| Na (ppm) | 0.85 | 0.009 |
| Fe (both ions, ppm) | 0.70 | 0.039 |
| Mn (ppm) | 0.69 | 0.042 |
| Mg (ppm) | 0.14 | 0.462 |
| Ca (ppm) | 0.13 | 0.480 |
| SO_4_^2-^ (mg/L) | 0.78 | 0.020 |
| Fe^2+^ (mg/L) | 0.32 | 0.237 |
| NO_3_^-^ (mg/L) | 0.21 | 0.366 |
| DO (mg/L) | 0.04 | 0.687 |
| H_2_ (mg/L) | 0.03 | 0.739 |
| S^2-^ (μg/L) | 0.02 | 0.790 |
| NH_4_^+^ (mg/L) | 0.01 | 0.880 |
